# Pathogenesis of DJ-1/PARK7-Mediated Parkinson’s Disease

**DOI:** 10.3390/cells13040296

**Published:** 2024-02-06

**Authors:** Line Duborg Skou, Steffi Krudt Johansen, Justyna Okarmus, Morten Meyer

**Affiliations:** 1 Department of Neurobiology Research, Institute of Molecular Medicine, University of Southern Denmark, 5230 Odense, Denmark; duborg@health.sdu.dk (L.D.S.); steffijohansen@health.sdu.dk (S.K.J.); jokarmus@health.sdu.dk (J.O.); 2 Department of Neurology, Odense University Hospital, 5000 Odense, Denmark; 3 BRIDGE—Brain Research Inter-Disciplinary Guided Excellence, Department of Clinical Research, University of Southern Denmark, 5000 Odense, Denmark

**Keywords:** Parkinson’s disease, *PARK7*, DJ-1, neuroprotection, neurotoxicity, mitochondria, oxidative stress, reactive oxygen species

## Abstract

Parkinson’s disease (PD) is a common movement disorder associated with the degeneration of dopaminergic neurons in the substantia nigra pars compacta. Mutations in the PD-associated gene *PARK7* alter the structure and function of the encoded protein DJ-1, and the resulting autosomal recessively inherited disease increases the risk of developing PD. DJ-1 was first discovered in 1997 as an oncogene and was associated with early-onset PD in 2003. Mutations in DJ-1 account for approximately 1% of all recessively inherited early-onset PD occurrences, and the functions of the protein have been studied extensively. In healthy subjects, DJ-1 acts as an antioxidant and oxidative stress sensor in several neuroprotective mechanisms. It is also involved in mitochondrial homeostasis, regulation of apoptosis, chaperone-mediated autophagy (CMA), and dopamine homeostasis by regulating various signaling pathways, transcription factors, and molecular chaperone functions. While DJ-1 protects neurons against damaging reactive oxygen species, neurotoxins, and mutant α-synuclein, mutations in the protein may lead to inefficient neuroprotection and the progression of PD. As current therapies treat only the symptoms of PD, the development of therapies that directly inhibit oxidative stress-induced neuronal cell death is critical. DJ-1 has been proposed as a potential therapeutic target, while oxidized DJ-1 could operate as a biomarker for PD. In this paper, we review the role of DJ-1 in the pathogenesis of PD by highlighting some of its key neuroprotective functions and the consequences of its dysfunction.

## 1. Introduction

Parkinson’s disease (PD) is a chronic and progressive neurodegenerative disease that was first described medically in 1817 by James Parkinson as “shaking palsy” [1]. Since then, PD has been known as a movement disorder, affecting millions of people worldwide. Patients present with motor symptoms, such as resting tremor, bradykinesia, rigidity, impaired posture and balance, and altered reflexes. PD also presents with non-motor symptoms, such as constipation, sleep-cycle disruptions, and cognitive dysfunction. Non-motor symptoms may occur years before diagnosis but can be difficult to assign to PD because of their generality [2,3].

The pathology of PD is characterized by the degeneration of dopaminergic neurons in the substantia nigra pars compacta (SNc) and the accumulation of Lewy bodies (LB) [4]. LB are protein aggregations of 100 different proteins, where the main component is alpha-synuclein (α-syn). These protein aggregates present in the cytoplasm and disrupt cell function by displacing other cell components and impairing mitochondrial, lysosomal, and proteasomal functions [5,6]. LB are also seen in other neurodegenerative diseases [7,8].

PD is an age-related disease. The average onset for sporadic PD is 60 years of age, and incidence increases with age. Other risk factors include pesticides, drugs, and environmental toxins [9,10]. Most cases of PD (90–95%) are of a sporadic onset, but 5–10% are hereditary with early onset, sometimes as low as 20 years of age [11]. Early-onset PD (EOPD) presents with symptoms similar to those of sporadic PD but shows some pathological differences. Familial PD is caused by mutations in D-related genes (PARK-genes) that are autosomal inherited and can raise the risk of developing PD. So far, 23 PARK-genes have been linked to PD [12,13,14]. These include *SNCA* (also known as *PARK1*/*4*) encoding α-syn; *PRKN* (*PARK2*) encoding Parkin; *PINK1* (*PARK6*) encoding PTEN-induced putative kinase 1; and *PARK7* encoding Parkinson’s Disease Protein 7 (DJ-1) [15]. A common feature of these genes is their important role in maintaining and regulating cellular and mitochondrial function and quality control [15,16].

DJ-1 is a multifunctional protein encoded by the gene *PARK7* that was first discovered in 1997 and described as an oncogene by Nagakubo et al. The group found that DJ-1 showed cooperative transforming activity with H-Ras in mouse NIH3T3 cells [17]. The link between DJ-1 and PD was described in 2003 by Bonifati et al., when a Dutch family and an Italian family presenting with early-onset parkinsonism were found to have mutations in the *PARK7* gene [18]. The Dutch family had a homozygous deletion that resulted in no expression of DJ-1, while the Italian family had a homozygous point mutation that led to the expression of non-functional DJ-1 [18]. Mutations in the protein DJ-1 cause autosomal recessive forms of PD, but oxidized DJ-1 is also found in the brains of idiopathic PD individuals [19,20].

**Figure 1:**
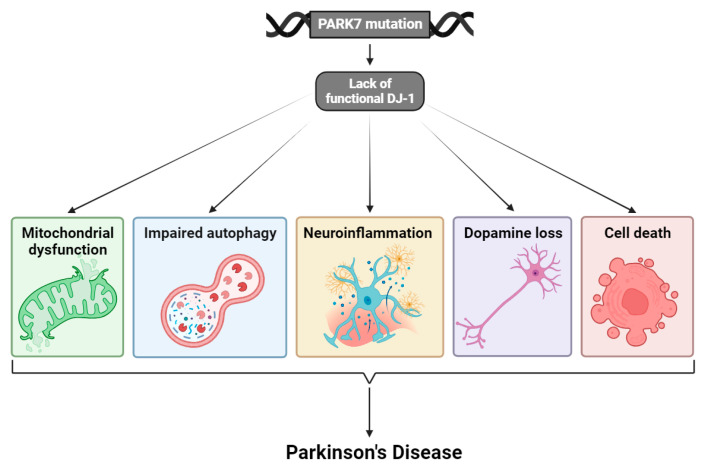
Mutations in the *PARK7* gene lead to transcription of dysfunctional DJ-1 protein. This has fatal consequences for the cells, and neurons in particular, and results in mitochondrial dysfunction, impaired autophagy, neuroinflammation, loss of the neurotransmitter dopamine, and cell death. The interplay between all these processes and factors is contributing to the pathogenesis of PD. Illustrated with BioRender.com.

Increasing evidence indicates that oxidative stress, and inability to suppress this development, is an important mediator of PD pathogenesis [21]. Energy failure as a result of mitochondrial dysfunction, impaired autophagy, neuroinflammation, loss of the neurotransmitter dopamine, and cell death have also been suggested as key factors in the pathogenesis of PD (Figure 1) [16,22,23]. DJ-1 is especially involved in the regulation of oxidative stress and mitochondrial function [24,25].

In this review, we describe the multiple functions of DJ-1 protein and discuss how its loss-of-function affects the pathogenesis of PD.

## 2. Structure and Post-Translational Modification of DJ-1 Protein

The DJ-1 gene maps to chromosome 1p36 [26]. DJ-1 comprises 189 amino acids and is an evolutionarily conserved protein with considerable sequence homology, with both the ThiJ family of bacterial proteins implicated in thiamin synthesis and the PfpI family of intracellular proteases [27]. The monomeric DJ-1 contains eight α-helices and eleven β-strands that are organized into a helix–strand–helix sandwich [28,29]. Endogenously expressed DJ-1 is found in a variety of tissues throughout the body but is highly expressed in the brain [30]. It is present in both neurons and glia (especially in astrocytes in the frontal cortex and substantia nigra) in patients with DJ-1 PD and in healthy brains [31]. Under basal conditions, DJ-1 is localized largely in the cytoplasm but also the mitochondria and nucleus [32]. DJ-1 contains three cysteine residues at positions 46, 53, and 106 amino acids from the N-terminal [33]. Oxidation of Cys-106 does not change the overall structure of DJ-1 significantly, but it can change the local structure and, thus, affect the stability and function of the protein. This change greatly increases the possibility of disease onset [34].

**Figure 2:**
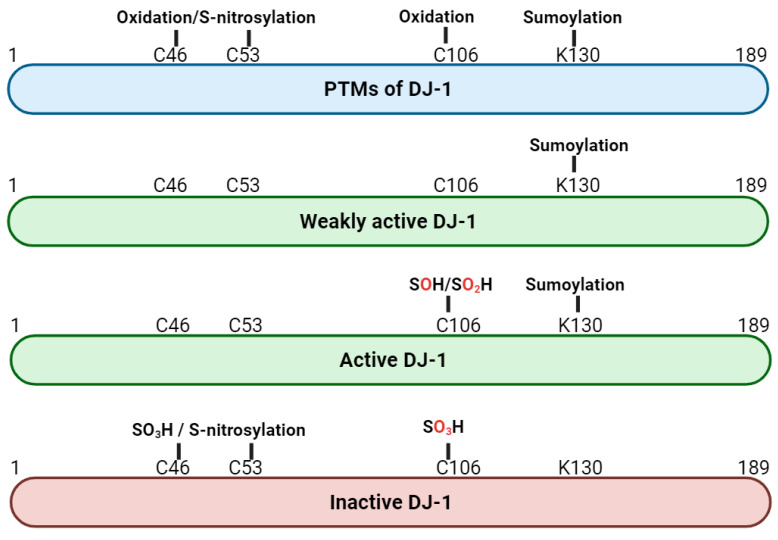
Post-translational modifications (PTMs) of DJ-1, including oxidation at cysteine residues C46, C53, C106, sumoylation at lysin residue K130, and S-nitrosylation at cysteine residues C46 and C53. The activity of DJ-1 changes depending on the PTM. The oxidations SOH and SO_2_H of C106 and sumoylation of K130 lead to an active DJ-1, whereas the oxidation to SO_3_H of C106, excessive sumoylation of K130, and S-nitrosylation result in an inactive form of DJ-1. Illustrated with BioRender.com.

DJ-1 can undergo various post-translational modifications (PTMs) (Figure 2). The cysteine residue C106 can be oxidized in three different states: SOH, SO_2_H, and SO_3_H. SOH and SO_2_H are thought to be active forms of DJ-1, whereas SO_3_H is thought to inactivate DJ-1. C106 is a highly sensitive sensor of oxidative stress, and an excess of oxidized DJ-1 has been found in brains of patients with PD [35,36]. In addition to oxidation, DJ-1 can undergo sumoylation at lysine 130 (K130) and S-nitrosylation at cysteine 46 (C46) and 53 (C53). Sumoylation of K130 is necessary for DJ-1′s activity and occurs in response to oxidative stress. Excess sumoylation inactivates DJ-1 and is seen in L166P mutants [37]. S-nitrosylation occurs under nitrosative stress and affects DJ-1′s ability to dimerize, resulting in an inactive form of DJ-1 [38].

## 3. Mutations of DJ-1

The activity or relevance of a given protein is sometimes first discovered or acknowledged when the exact cell function of this protein is still unknown. The function of DJ-1 was suggested due to inactivating mutations [26]. Bonifati et al. discovered that the Dutch family with parkinsonism lacked DJ-1 due to a deletion of several exons in the *PARK7* gene, while the Italian family had a single point mutation, L166P, in the *PARK7* gene. The leucine residue at position 166 substituted with a proline is highly conserved, and this mutation results in functionally inactive DJ-1 [18]. Based on the crystal structure of DJ-1, it was later hypothesized that the inactivity related to the L166P mutation was caused by destabilization of DJ-1′s dimer interface [39].

Since then, more mutations in the *PARK7* gene have been found to interrupt the dimeric structure of DJ-1. The protein is active as a homodimer, but the mutations E64D, M26I, and D149A have also been shown to form heterodimers with wildtype DJ-1, resulting in inactivation of the protein [18,40,41]. As residue C106 is thought to be most important in relation to DJ-1 activity, its mutation effects have been studied. While point mutation C106A prevents oxidation of C106, point mutation C106D mimics sulfonic oxidative modification of C106, which is the oxidative state where DJ-1 is thought to be active [33,42]. Although the “phenotype” is similar, the distribution of the C106D mutant is different from that of wildtype DJ-1 and impairs the function of DJ-1 [18,43].

Although gain-of-function mutations in the PARK7 gene have been reported and related to various types of cancer, it is the loss-of-function mutations in the gene that have been related to autosomal recessive EOPD [17,44,45].

## 4. The Multiple Cellular and Molecular Functions of DJ-1

**Figure 3:**
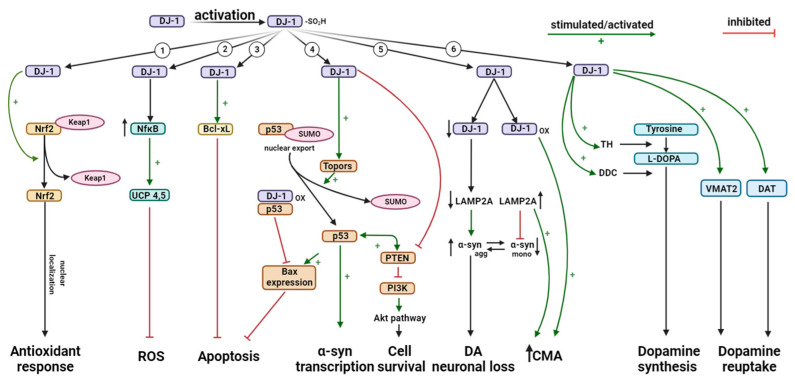
Cellular effects of the *PARK7*-encoded protein DJ-1. The multifunctional protein DJ-1 plays a role in many cellular systems, including (1) the endogenous antioxidant system by activating Nrf2 through dissociation from its inhibitor Keap1, (2) inhibition of reactive oxygen species (ROS) production by acting as transcriptional coactivator of NfkB and subsequent transcription of the genes encoding UCP4,5, (3) inhibition of apoptosis through upregulation and stabilization of Bcl-xL in mitochondria, (4) positive regulation of proapoptotic p53 transcriptional activity through Topors-mediated sumoylation (SUMO), leading to increased Bax expression and a-synuclein transcription, and direct repression with subsequent suppression of Bax-mediated apoptosis. DJ-1 also inhibits PTEN, activating the PI3K/Akt pathway resulting in cell survival, and inhibiting p53-induced apoptosis, (5) regulation of the activity of chaperone-mediated autophagy (CMA), LAMP2A levels, and a-synuclein aggregation, and (6) stimulation of dopamine synthesis via activation of tyrosine hydroxylase (TH) and 4-dihydroxy-L-phenylalanine decarboxylase (DDC). Furthermore, DJ-1 stimulates the expression of the dopamine transporters VMAT2 and DAT, resulting in dopamine reuptake that decreases dopamine toxicity. Illustrated with BioRender.com.

Since the linking of DJ-1 to autosomal recessive EOPD in 2003 [18], the protein has been extensively studied and appears to have several functions within the cell. It was clear that DJ-1, or more precisely the lack of its activity, had pathogenic consequences in the development of PD [20,21,22,23]. But what is the exact cellular function of DJ-1? This question has been of interest throughout the last 25 years. Some of the various functions DJ-1 exerts include transcriptional regulation, oxidative stress reaction, reactive oxygen species (ROS) scavenging, and chaperone, protease, and mitochondrial regulation (Figure 3) [27,32,33,36,46,47,48]. More recent research points towards DJ-1 also having a regulatory role in immune and inflammatory responses [49].

### 4.1. Role of DJ-1 in Mitochondrial Homeostasis

Mitochondrial homeostasis is crucial for cell homeostasis and is ensured through mitochondrial quality control (MQC). MQC covers several mechanisms: mitochondrial fusion and fission, mitophagy, and mitochondrial biogenesis [50]. Alterations in mitochondrial morphology and homeostasis and calcium homeostasis can have a tremendous impact on neuronal synaptic activity and survival, and mitochondrial dysfunctions have been linked to PD and to aging in general [51,52].

As mentioned earlier, DJ-1 is mainly present in the cell cytoplasm, and only a small portion resides in mitochondria [32]. However, DJ-1 translocates to the outer mitochondrial membrane (OMM) upon oxidative stress [17,53]. This translocation is believed to be a consequence of residue C106′s sensitivity to ROS, since the oxidation of C106 activates DJ-1 and initiates translocation to the OMM [54]. There are different suggestions regarding the interaction of DJ-1 with the mitochondria. As DJ-1 binds to subunits of mitochondrial complex I and was found to interact with mitochondrial DNA-encoding proteins that are synthesized inside the cell using mitochondrial ribosomes, it was concluded that DJ-1 interacts with mitochondrial complex I both at the OMM and in the mitochondria [55,56]. Studies in DJ-1 knockout (KO) mice and cells showed impaired complex-I-dependent respiration, and DJ-1 KO produced changes in mitochondrial morphology that were linked to mitochondrial ROS production and impaired respiration [55,56]. This impairment could decrease the energy outcome of cell metabolism, which is especially problematic for dopaminergic neurons that require high amounts of energy due to their axonal connections with other areas involved in movement and motor control [57,58,59]. Humans carrying the homozygote E64D mutation in DJ-1 showed the same dysfunctional mitochondrial phenotype as in DJ-1 KO mice, indicating that DJ-1 plays a crucial role in conserving mitochondrial morphology in PD [55].

#### Interplay of Parkin, PINK1, and DJ-1

Mitophagy is an autophagy pathway selective for mitochondria. The process degrades dysfunctional mitochondria damaged by oxidative stress. Mitophagy is crucial in maintaining MQC, so it is important that mechanisms affecting this process are not disrupted [60,61]. Various pathways are assigned to mitophagy, but the best described involves two PARK-genes, PINK1 and Parkin, and is called the PINK1-dependent Parkin-mediated autophagy [62,63].

**Figure 4:**
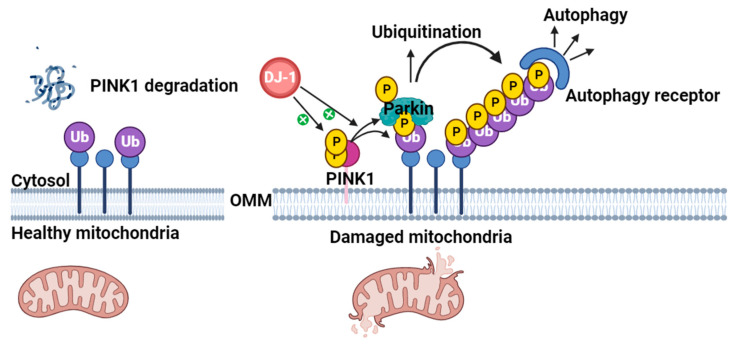
The PINK1/Parkin pathway in mitochondrial quality control (MQC). When the outer mitochondrial membrane (OMM) is functional, PINK1 is degraded in the cytosol. When the OMM is disrupted, PINK1 is recruited to the OMM where it accumulates, is activated, and auto-phosphorylates, thus facilitating Parkin ubiquitination (Ub) and mitophagy of the damaged mitochondrial membrane proteins. Ubiquitination by Parkin enhances mitophagy, whereas dysfunction of Parkin causes accumulation of substrates and proteins otherwise marked for degradation. DJ-1 is thought to enhance the activity of PINK1 at the OMM, thereby supporting the PINK1-dependent Parkin-mediated mitophagy, but the exact mechanism is not clear. Illustrated with BioRender.com.

Like DJ-1, PINK1 is a protein encoded by a gene associated with EOPD. When present in the cytosol, PINK1 is believed to be involved in the differentiation of neurons [64]. PINK1 is also localized at the OMM. In a functional mitochondrion, PINK1 is degraded in the cytosol due to the mitochondrial membrane potential (MMP) [65]. In damaged or dysfunctional mitochondria, the MMP is altered and PINK1 accumulates on the surface of the OMM. This accumulation activates PINK1, which auto-phosphorylates and recruits Parkin to the mitochondria. Parkin ubiquitinates the damaged mitochondrial membrane proteins, which can then be degraded through mitochondrial autophagy (Figure 4) [66,67]. Loss of PINK1 has been associated with mitochondrial defects and degeneration of dopaminergic neurons in *Drosophila* [68], while dysfunction of Parkin causes accumulation of substrates and proteins that would otherwise be marked for degradation by Parkin [69]. In 2010, several studies established that the Parkin-mediated mitophagy is dependent on PINK1 [70,71,72].

As DJ-1 also translocates to the OMM upon oxidative stress [54], it has been studied whether the three proteins participate in the same pathway. In 2006, Tang et al. showed that DJ-1 and PINK1 associate and form a complex that protects cells against oxidative stress [73]. Both mutant and wildtype forms of DJ-1 and PINK1 interacted with each other, probably through the N-terminal part of PINK1′s kinase domain. Wildtype DJ-1 stabilized PINK1 in cells, whereas mutant forms of DJ-1 degraded PINK1, suggesting that DJ-1 could play a “supportive role” in PINK1-dependent Parkin-mediated mitophagy [73].

Although the interplay between DJ-1, PINK1, and Parkin remains unresolved, it is clear that DJ-1 plays an important role in mitochondrial homeostasis and maintenance of cellular health. Dysregulation of mitochondrial homeostasis enhances ROS production, highlighting the importance of DJ-1 in the prevention of PD progression.

### 4.2. Antioxidant Activity of DJ-1

Oxidative stress plays an important role in the degeneration of dopaminergic neurons and has been linked to progression of PD [21]. As with hydrogen peroxide (H_2_O_2_) and superoxide anion ($O_{2}^{-}$), excessive ROS production can be harmful to various cellular components and creates an imbalance between ROS levels and the antioxidant capacity of the cell during oxidative stress [33]. DJ-1 has been suggested to be an antioxidant protein that is important in the oxidative stress response [18,25,36,41,74,75]. This was shown to be correct in a study demonstrating the ability of DJ-1 to eliminate hydrogen peroxide by oxidizing itself [46]. Furthermore, the study found that mutations in DJ-1 led to oxidative-stress-induced cell death, indicating a protective role of DJ-1 [46]. When exposed to oxidative stress, some of the proteins located in the cytoplasm first translocate to mitochondria and afterward to the nucleus [32]. The oxidative modification of Cys-106 to the sulfinic acid form enhances the DJ-1 relocation to the mitochondria during oxidative stress and is, therefore, required for the protective function of DJ-1 [42]. However, another study found no need for oxidation of cysteine residues for DJ-1 to translocate [32].

DJ-1 is involved in the regulation of nuclear factor erythroid 2-related factor (Nrf2), one of the most important transcription factors for upregulating the expression of antioxidant genes [76]. Under normal conditions, Nrf2 is sequestered in the cytoplasm by binding to Kelch-like ECH-associated protein 1 (KEAP1). However, under conditions of oxidative stress, DJ-1 had been shown to interact with KEAP1, leading to the release and activation of Nrf2 (Figure 3) [77]. Once activated, Nrf2 translocates into the nucleus and binds to the antioxidant response element (ARE) in the promoter regions of various genes involved in antioxidant defense and detoxification. This includes genes encoding enzymes, such as heme oxygenase-1 (HO-1), NAD(P)H quinone oxidoreductase 1 (NQO1), and glutathione S-transferase (GST) [78,79]. It has been shown that deficiency of DJ-1 leads to unstable Nrf2 protein in both transformed human cell lines and primary cells, decreasing transcriptional responses [77,80]. Another study found silencing of DJ-1 to result in reduced Nrf2 expression and activity and increased ROS production; Nrf2 probably, thus, works downstream of DJ-1 as silencing of Nrf2 did not alter the expression of DJ-1 [81].

Oxidative stress in mitochondria will induce defenses that cause transient and mild mitochondrial depolarization or uncoupling [82]. In the inner mitochondrial membrane, uncoupling proteins (UCPs) transport protons from the intermembrane space to the matrix to prevent an excessive increase in mitochondrial membrane potential (MMP) and ROS development, two products of oxidative phosphorylation [83]. DJ-1 protects the cell from oxidative stress by regulating the transcriptional activity of UCP4 and UCP5 via the NFκB pathway, resulting in preservation of intracellular ATP and MMP (Figure 3) [84]. UCP4 and UCP5 are thought to play a key role in regulating calcium levels in mitochondria, and DJ-1 knockout (KO) results in downregulation of UCP4 and UCP5 expression, impaired function of calcium-induced uncoupling, and increased oxidation of matrix proteins in dopaminergic neurons of the SNc. Furthermore, DJ-1 modulates the magnitude of the UCP4- and UCP5-mediated response to oxidative stress, suggesting an important role of DJ-1 in regulating mitochondrial mechanisms [83,84,85].

Overall, DJ-1 is an important redox-reactive signaling intermediate that controls oxidative stress during PD-related neurodegenerative processes. Its loss of function, thus, leads to the progression of PD.

### 4.3. Role of DJ-1 in Inflammatory Responses

In addition to the functions of DJ-1 described above, it has also been shown to interfere with inflammatory responses through its regulation of ROS levels [49,86]. Although excess ROS production in cells is toxic in immunological terms, appropriate levels of ROS can initiate signal transduction that can regulate inflammatory reactions in the host [87,88,89,90]. DJ-1 overexpression has, moreover, been found to suppress the production of proinflammatory cytokines, such as interleukin-6 (IL-6) and tumor necrosis factor-alpha (TNF-α), suggesting a potential protective role [91,92]. Nuclear factor-kappa B (NF-κB) is a key transcription factor involved in the regulation of inflammatory gene expression. DJ-1 has been reported to interact with NF-κB and modulate its activity. The interaction between DJ-1 and NF-κB is thought to influence the transcriptional activity of NF-κB, therby affecting the expression of inflammatory mediators [93].

In the context of neuroinflammation, DJ-1 has been suggested to regulate microglial activation, influencing the release of inflammatory molecules. The dysregulation of microglial activation is implicated in neuroinflammatory processes associated with neurodegenerative diseases, such as PD [22].

While the anti-inflammatory properties of DJ-1 are recognized, the precise molecular mechanisms through which the protein modulates inflammatory responses are not fully understood. Further research is needed to elucidate the detailed pathways and interactions involved.

### 4.4. Role of DJ-1 in Apoptosis

In mitochondria, the protein Bcl-XL has a critical role in protecting cells against death. If exposed to ultraviolet B (UVB) irradiation, DJ-1 will translocate into the mitochondria and interact with Bcl-XL. The interactions between DJ-1 and Bcl-XL are dependent on oxidation [94]. Only the oxidized form of DJ-1 will bind to Bcl-XL, predominantly in the hydrophobic groove surrounded by the domains BH1-BH3. The C-terminal 8-helix peptide of DJ-1 acts as a Bcl-XL-binding motif by binding to the pro-apoptotic BH3 peptide-binding hydrophobic groove in Bcl-XL [95]. By binding to Bcl-XL, DJ-1 stabilizes the protein (Figure 3) by inhibiting the ubiquitination and degradation of Bcl-XL through the ubiquitin protease system initiated by the UVB irradiation. However, KD of DJ-1 will prevent the stabilization and lead to the ubiquitination and degradation of UVB-irradiated Bcl-XL [94].

DJ-1 also regulates the pro-apoptotic transcription factor p53 through Topors-mediated sumoylation. DJ-1 binds to Topors/p53BP3, a ring finger protein that binds to both p53 and topoisomerase I (Figure 3). Both p53 and DJ-1 are sumoylated, where small ubiquitin-like modifiers are added to the proteins in a post-translational modification process. In vitro and in vivo studies found DJ-1 to be bound to p53, with its binding and colocalization stimulated by ultraviolet irradiation. The sumoylation of p53 by Topors abrogated the transcriptional activity of p53 but DJ-1 restored its repressed activity by releasing the sumoylated form of p53 [96]. Sumoylation of DJ-1 is critical for its function as a repressor of p53. By repressing p53 transcriptional activity, DJ-1 also decreases the expression of Bax, which is a transcriptional target of p53 and a regulator of apoptosis [97]. As a result, caspase activation is abolished, and cell death is avoided [98] (Figure 3). Regarding the association between DJ-1 and p53, the oxidation status of Cys-106 is also crucial for the activity level of DJ-1 [99].

Another effect of DJ-1 on p53 is the regulation of Sirtuin 1 (SIRT1). SIRT1 deacetylates p53, which is stimulated by DJ-1. The transcriptional activity of p53 is consequently suppressed, and apoptosis is prevented. KO of DJ-1 leads to reduced activity of SIRT1—an activity that could be restored by the reintroduction of wildtype DJ-1 but not Cys-106-mutated DJ-1 [100]. In a further study, the expression levels of acetylated p53 were also downregulated when overexpressing DJ-1 [88].

A transcriptional target of p53 is the PD-associated α-syn. p53 interacts physically with the promoter of α-syn, both in vitro and in physiological contexts. If p53 expression is upregulated, strong increases are observed in α-syn protein, promoter activity, and mRNA levels. In contrast, the depletion of endogenous p53 downregulates α-syn transcription. Furthermore, p53 DNA-binding dead mutations inhibit the p53 regulation of α-syn [101].

Another pathway that DJ-1 modulates is the phosphatase and tensin homolog (PTEN) phosphatidylinositol 3-kinase (PI3K)-Akt pathway through the regulation of the activity of PTEN that has a major role in neuronal cell death. A nitric oxide (NO) positioned on Cys-106 is transferred from S-nitrosylated DJ-1 to PTEN through transnitrosylation [102]. Consequently, the phosphatase activity of S-nitrosylated PTEN is decreased, which promotes cell survival by inhibiting the antagonizing effect of PTEN on PI3K and, thus, also the downstream neuroprotective Akt [102,103]. During nitrosative stress, DJ-1 thus detoxifies NO by transnitrosylation of PTEN, exerting neuroprotective qualities [102].

**Figure 5:**
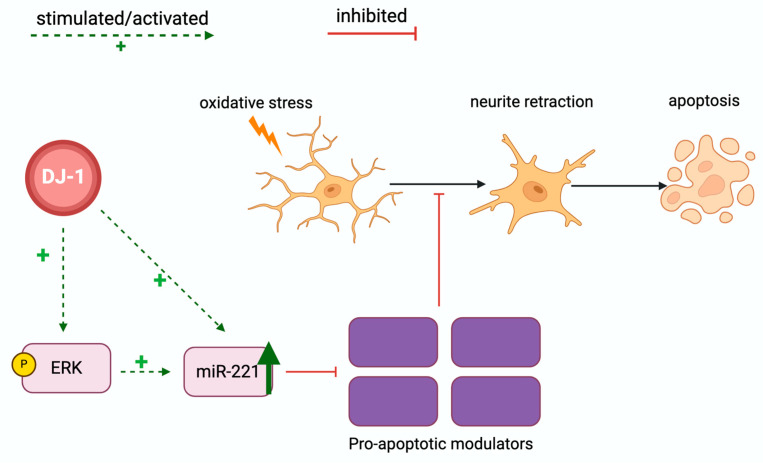
DJ-1 regulates apoptosis by increasing miR-221 expression. DJ-1 stimulates the phosphorylation of extracellular signal-regulated kinase (ERK), thus increasing ERK pathway activity. This increases miR-221 expression, which inhibits pro-apoptotic modulators from inducing apoptosis in neurons upon oxidative stress. DJ-1 also stimulates the expression of miR-221 directly. Illustrated with BioRender.com.

It has been suggested that dysregulation of miRNA is involved in the pathogenesis of PD [104,105]. In 2018, Oh et al. found that miR-221 was downregulated in both DJ-1 KD cells and murine brain tissue. Re-introducing DJ-1 restored the expression of miR-221, suggesting that DJ-1 and miR-221 are linked. The authors suggested that DJ-1 may regulate miR-221 expression partly through the extracellular signal-regulated kinase (ERK) pathway, resulting in cytoprotection of neuronal cells (Figure 5) [106].

In summary, DJ-1 is an essential regulator of apoptotic mediators, such as pro-apoptotic transcription factor p53 and anti-apoptotic protein Bcl-XL. Loss of this regulatory function increases cellular death and neuron loss, which are hallmarks of PD.

### 4.5. DJ-1 Regulates Degradation of Monomeric α-Synuclein (α-syn) via Chaperone-Mediated Autophagy (CMA)

α-syn is a protein that is highly associated with PD. Under physiological conditions, it is a neuronal protein that is involved in the regulation of synaptic vesicle trafficking and neurotransmitter release. It is a soluble, unfolded protein that accumulates in PD and is the main component of LB [107,108].

**Figure 6:**
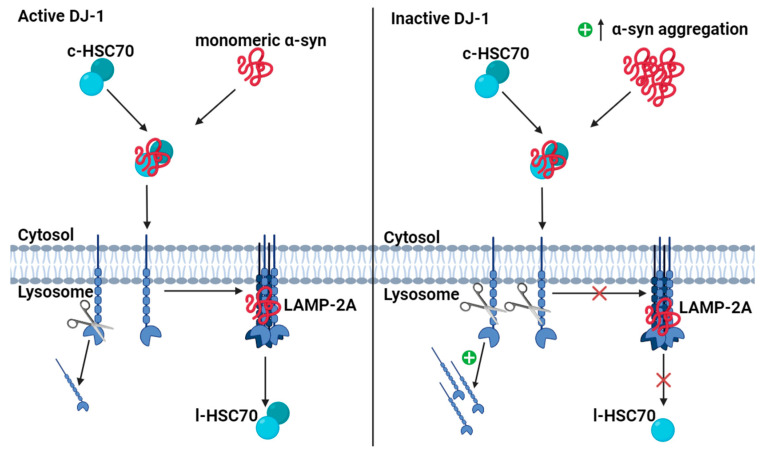
Degradation of monomeric α-synuclein (α-syn) via chaperone-mediated autophagy (CMA). Monomeric α-syn is degraded by LAMP-2A receptors in CMA. In the presence of DJ-1, the chaperone protein c-HSC70 binds monomeric α-syn in the cytosol and transports it to the lysosomal membrane, where the complex binds to a LAMP-2A monomer. When the complex is bound, LAMP-2A forms a functional homotetrameric receptor that allows α-syn to be translocated to the lysosomal lumen where it is degraded by proteases. HSC70 is reused in the lysosome as lysosomal HSC70 (l-HSC70). When DJ-1 is absent, monomeric LAMP-2A is degraded faster, and LAMP-2A receptors do not form as often. Consequently, monomeric α-syn cannot be degraded at the lysosomal lumen and instead accumulates in the cytosol. Illustrated with BioRender.com.

Wildtype monomeric α-syn is mainly degraded through chaperone-mediated autophagy (CMA) [109,110,111] (Figure 6). In the presence of active DJ-1, the chaperone protein heat-shock cognate protein of 70 kDa (HSC70) recognizes and binds monomeric α-syn in the cytosol along with co-chaperones. Upon binding, cytosolic HSC70 (c-HSC70) delivers the monomeric α-syn to the lysosomal membrane. The HSC70–substrate complex is recognized by the lysosome-associated membrane protein type 2A (LAMP-2A), which is the CMA receptor in the lysosomal membrane [109]. LAMP-2A exists as an inactive monomer in the lysosomal membrane, but upon HSC70–substrate complex binding, it forms a homotetramer. Only in this form is LAMP-2A active and α-syn can be translocated to the lysosomal lumen [112]. The activation of LAMP-2A is the rate-limiting step in CMA, and the level of lysosomal membrane LAMP-2A is directly related to the activity of CMA [113,114,115]. The translocation of α-syn is assisted by lysosomal HSC70 (l-HSC70), and after translocation, α-syn is degraded in the lysosomal lumen by proteases (Figure 6) [109,112]. In contrast, DJ-1 deficiency accelerates the degradation of LAMP-2A in lysosomes, leading to the aggregation of α-syn [23,113].

Another way that DJ-1 can influence the accumulation of α-syn is by regulating glycation through its suggested deglycase activity. DJ-1 is a deglycase protein and a major contributor to glycation damage repair [116]. Glycation is one of many non-enzymatic PTMs (ne-PTM). Methylglyoxal (MGO) is formed as a byproduct of glycolysis, the sugar metabolism that is highly important in the brain in view of the high energy demand, with sugar being the main substrate. MGO is a precursor of advanced glycation end-products (AGEs) that are associated with several different diseases [117]. Furthermore, MGO reacts with several proteins, DNA, and other biomolecules [118]. One of these is the glycation of the glyceraldhyd-3-phosphate dehydrogenase (GAPDH) enzyme, involved in glycolysis, resulting in reduced activity of GAPDH. MGO also glycates α-syn [119]. Interaction between glycated α-syn and GAPDH results in the inactivation of GAPDH, leading to the accumulation of metabolic byproducts, increased production of MGO, and cellular stress [120,121]. Newer research has shown that DJ-1 has an impact on the glycating agent, MGO. MGO is known to glycate α-synuclein at the N-terminal, which induces the formation of oligomers, promoting the formation of LB [121,122]. In 2021, Atieh et al. reported findings of DJ-1′s role in the glycation of α-syn, showing that DJ-1 scavenged for aggregated α-syn. The group proposed that DJ-1 suppresses the interaction between glycated α-syn monomers and oligomers and releases glycated monomeric α-syn in solution [122]. This interaction might prevent the formation of toxic oligomers, both from α-syn aggregates and from the high-glucose metabolism activity in neurons. The chaperone and deglycating activity of DJ-1 is still discussed. Reports suggest that the alleged deglycase activity of DJ-1 is actually due to its glyoxylase activity on MGO. Some studies suggest that it is the substrate that determines whether DJ-1 has deglycase or glyoxylase activity [116,123].

Since LB are a hallmark of PD and mainly consist of accumulated α-syn, it is highly relevant that the aggregation of monomeric α-syn is inhibited to prevent the progression of PD. DJ-1 contributes to this inhibition through enhanced CMA by upregulating LAMP-2A and enhancing autophagic reductions in α-syn monomers. As a consequence of the dysfunctional DJ-1 protein, decreased CMA activity enhances the accumulation of α-syn, leading to an elevated risk of LB formation and, thereby, PD progression.

### 4.6. DJ-1 Regulates Dopamine Homeostasis

**Figure 7:**
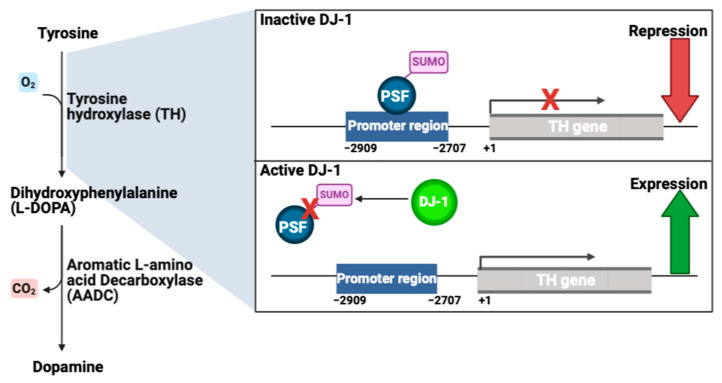
DJ-1 regulates the synthesis of dopamine. On the left is the synthesis of dopamine from tyrosine. Tyrosine is converted to L-DOPA by tyrosine hydroxylase (TH). L-DOPA is then converted to dopamine by aromatic L-amino acid decarboxylase (AADC). On the right is an illustration of the regulatory function of DJ-1 on the promotor region of the human TH-gene. Without DJ-1 present, the co-repressor pyrimidine tract-binding protein-associated splicing factor (PSF) is sumoylated and binds to the promoter region of the TH-gene, inhibiting the expression of TH. When DJ-1 is present, the sumoylation of PSF is blocked and it cannot therefore bind the promoter region of the TH-gene, resulting in transcription of TH. Hence, DJ-1 increases expression of human TH-gene on transcriptional level, leading to higher amounts of TH for dopamine synthesis. Illustrated with BioRender.com.

Dopamine is synthesized from tyrosine in a two-step reaction. Tyrosine is transformed to dihydroxyphenylalanine (L-DOPA) by tyrosine hydroxylase (TH), which is the rate-limiting step of the synthesis. Aromatic L-amino acid decarboxylase (AADC) then converts L-DOPA to dopamine [124]. DJ-1 blocks the sumoylation of pyrimidine tract-binding protein-associated splicing factor (PSF). PSF acts as a co-repressor and, therefore, inhibits the expression of TH when bound to the promotor region of the gene. When DJ-1 is present, PSF is inactivated and cannot bind the promotor region of TH. This enables the binding of a co-activator to the promoter region, whereby transcription of the TH-gene is initiated (Figure 7) [79,125].

In addition to regulating the synthesis of dopamine on a transcriptional level, DJ-1 also affects the distribution and availability of dopamine by regulating the expression of vesicular monoamine transporter 2 (VMAT2) in dopaminergic neurons (Figure 7). Regulation of dopamine reuptake decreases the risk of dopamine toxicity, avoiding loss of dopaminergic neurons [49,126,127,128]. Hence, DJ-1 is an important regulator of not only the synthesis of dopamine but also its reuptake and, therefore, the availability of dopamine. Dysregulation of these processes is known to cause some of the central features of PD.

## 5. DJ-1 as a Biomarker and Therapeutic Agent

It has been proposed that oxidized DJ-1 could be used as a biomarker in the detection of PD. Not only do mutations in DJ-1 cause autosomal recessive PD, but DJ-1 is implicated in the sporadic form of PD. One study found oxidatively damaged DJ-1 in the brains of patients with idiopathic PD [129]. Compared to medicated patients with PD and healthy controls, the erythrocytes of unmedicated patients with PD contained significantly more oxidized DJ-1. Specific antibodies against Cys-106-oxidized DJ-1 have been developed and suggested as a potential application for the early diagnosis of PD [130]. One study even found 2-fold higher levels of oxidized DJ-1 in the urine of Korean patients with PD than in healthy controls [131]. In addition to oxidized DJ-1, the reduced form of DJ-1 could be relevant to detect. This would enable the determination of both oxidized DJ-1 and the ratio between it and the reduced form, thus providing more information than the level of oxidized DJ-1 alone. A chemical probe capable of monitoring reduced DJ-1 has been identified in situ, making it possible to detect the ratio [132]. Although the results of these studies appear promising for oxidized DJ-1 as a biomarker for PD, further studies are needed for validation, especially with larger cohorts of both patients with PD and healthy controls.

With its definite involvement in the pathogenesis of PD, DJ-1 is a potential therapeutic target. As DJ-1 exhibits neuroprotective roles, one attempted approach has been to simply increase the levels of DJ-1. The overexpression of recombinant DJ-1 in PD rats was effective in reducing the death of dopaminergic neurons in the substantia nigra. However, the rats were injected intranigrally, which is far from applicable in humans [133,134]. One way to overcome this obstacle could be the use of cell-permeable peptides that can cross the cellular plasma membrane and conveniently deliver biologically active proteins intracellularly to all tissues throughout the body, including the brain. TAT is a membrane-permeable carrier peptide vector that is acquired from the transduction domain from the human immunodeficiency virus [135]. By fusing TAT with DJ-1, 6-hydroxydopamine, toxicity was reduced in vivo in mice [136].

Another therapeutic approach is to prevent the harmful and inactivating excessive oxidation of Cys-106 in DJ-1. Compounds that can bind to the Cys-106 region have already been identified and isolated. These compounds can prevent oxidative-stress-induced death of a variety of cells, with effects specific to DJ-1. Furthermore, they inhibited the production of ROS, restored the lost activity of mitochondrial complex I and tyrosine hydroxylase, and prevented dopaminergic cell death in the substantia nigra of PD model rats [137]. In particular, compound-23 has proven to be effective in restoring neuronal cell death in substantia nigra and striatum; it also restores locomotion defects in neurotoxin-induced PD mice models [138]. The development of therapies that will inhibit oxidative-stress-induced neuronal cell death is critical as the current therapies for PD treat only its symptoms.

## 6. Discussion

Despite immense efforts to elucidate the exact role of DJ-1 in the pathogenesis of PD, it is not yet completely clear. The abovementioned functions of DJ-1 have expanded our knowledge of the protein, but they are probably only part of the overall picture and have not led to a complete understanding of the molecular mechanisms behind PD. We have a much-improved understanding of the pathogenic mechanisms and especially the importance of the role of DJ-1 in oxidative stress, but although DJ-1 works as a scavenger of ROS, the effectiveness of this specific role has been questioned. The activity of DJ-1 is much slower than that of the standard peroxiredoxin found in humans, and DJ-1 is not catalytically converted back to its reduced state. This suggests that the most important role of DJ-1 as an antioxidant is not as an ROS scavenger but presumably as a binder and regulator of other proteins that can then exert their own functions, as, for example, transcription factors in diverse signaling pathways [139,140].

Various theories have been suggested for the many roles and functions of DJ-1. One theory relates to the ability of DJ-1 to regulate miRNA transcriptionally in many tissues other than the brain. Increased levels of different miRNAs downregulate DJ-1 levels, which supports a connection between DJ-1 and miRNAs. It seems that levels of miRNAs and their effect on DJ-1 activity differ in different parts of the brain [141]. Another theory suggests that DJ-1 is a moonlighting protein, i.e., a type of multifunctional protein with multiple biochemical or biophysical functions that are not due to gene fusions or multiple RNA splice variants [142]. As DJ-1 is a member of the ThiJ/DJ-1/PfpI protein superfamily (which is a moonlighting protein family), this may be a plausible argument. Members of the ThiJ/DJ-1/PfpI superfamily exert numerous functions to manage cellular stress [143] and are highly evolutionary conserved. For example, Hsp31 has shown more activity towards α-syn toxicity than DJ-1 [144,145]. Although DJ-1 meets many of the moonlighting protein criteria, it has not yet been recognized as one itself.

## 7. Conclusions—Many Functions, but One Goal: Neuroprotectivity

Since its discovery in 1997 and original labeling as an oncogene, DJ-1 has been assigned a range of functions. It was later associated with EOPD and functions contributing to neuroprotection. Although *PARK7*-mediated PD is a rare variant of PD, it is still important to understand the underlying mechanisms of this disease. The function of DJ-1 opens many doors in the molecular understanding of PD as DJ-1 participates in mechanisms with other PD-related proteins.

It is now very clear that the main function of DJ-1 is to protect the neuron from oxidative stress through transcriptional regulation, chaperone activity, and initiation of mechanisms in response to cellular events, such as regulating dopamine synthesis. Newer research suggests its role in the inflammatory response, which is also an important factor in the pathogenesis of PD [49]. DJ-1 also seems to have a somewhat debated function, namely the ability to convert glycated proteins such as α-syn and products of the glucose metabolism [146,147]. Although DJ-1 is mainly associated with EOPD, it could also play a role in sporadic PD. DJ-1 is oxidized with aging, suggesting that the activity of DJ-1 and, thereby, its neuroprotective role might reduce with aging. This includes the stimulatory function towards the synthesis of dopamine and other factors in the progression of PD [148]. Altered activity of DJ-1 exposes cells to oxidative stress with impaired neuroprotective functions and increases the risk of developing PD.

Regardless of its specific function in a given situation, DJ-1 appears to have one primary goal: cellular health through protection of neurons from cellular damage. The evidence indicates that loss of DJ-1 impairs neuroprotective activity and increases vulnerability to PD [36].

## Data Availability

Not applicable.
